# Pain Management Strategies in Pediatric Dentistry: A Systematic Review of Clinical Approaches and Public Health Implications

**DOI:** 10.7759/cureus.106261

**Published:** 2026-04-01

**Authors:** Sujatha P, Shreya Patel, Pauline Susan Palose, Sowmya Akkanapally, Maryam Omer Shamim, Kinza Qureshi

**Affiliations:** 1 Department of Pediatric and Preventive Dentistry, Bharati Vidyapeeth (Deemed to be University) Dental College and Hospital, Sangli, IND; 2 Department of Dental Science, Dharmsinh Desai University, Nadiad, IND; 3 Department of Conservative Dentistry and Endodontics, Vinayaka Mission’s Sankarachariyar Dental College, Salem, IND; 4 Department of Dentistry, Panineeya Institute of Dental Sciences & Research Centre, Hyderabad, IND; 5 Department of Dentistry, Liaquat College of Medicine & Dentistry, Karachi, PAK; 6 Department of Prosthodontics, Rehmat Memorial Post Graduate Dental Hospital, Abbottabad, PAK

**Keywords:** behavior management, dental anxiety, pain management, pediatric dentistry, public health dentistry

## Abstract

Pain management is a fundamental aspect of pediatric dentistry (PD), as children’s dental experiences are closely linked to pain perception, anxiety, and subsequent oral health behaviors. Although numerous pharmacological, behavioral, and technology-assisted strategies have been explored, uncertainty persists regarding their combined effectiveness and wider public health relevance. This systematic review synthesized current evidence on pain management strategies in PD and evaluated clinical outcomes alongside implications for access, equity, and quality of care. A comprehensive literature search was conducted in PubMed/MEDLINE, Scopus, Web of Science, and the Cochrane Library, covering studies published between 2015 and 2025. Randomized controlled trials and observational comparative studies involving children aged zero to 18 years and reporting pain, anxiety, behavioral, or treatment acceptance outcomes during dental procedures were included following the PRISMA guidelines. After screening and eligibility assessment, 11 studies were included in the final qualitative synthesis. The evidence indicates that pharmacological interventions such as local anesthesia and sedation remain effective for controlling procedural pain, particularly during invasive treatments, while nonpharmacological and technology-assisted approaches reduce anxiety and improve cooperation. Multimodal strategies consistently demonstrated superior outcomes compared with single interventions. These findings highlight implications for patient-centered care, improved treatment acceptance, and reduced fear-related avoidance of dental services, particularly among vulnerable pediatric populations globally.

## Introduction and background

Pain is a major concern in pediatric dentistry (PD) because children encounter dental procedures through developing neurobiologic, cognitive, and emotional frameworks that increase sensitivity, influence fear, and cooperate, making effective control of pain fundamental to safe, ethical, and patient-centered dental healthcare delivery in all local and global settings today [1]. Across the scale of populations globally, untreated dental caries and invasive restorative treatments are still highly prevalent in childhood and often accompanied by pain, anxiety, and poor behavioral outcomes that can continue into adolescence and adulthood, compromising quality of life with respect to oral health and patterns of long-term service use across different systems [2]. Early dental experiences that include poorly managed discomfort often contribute to the development of dental fear, anticipatory anxiety, and avoidance behaviors that lead to the clinical challenge of dental management, decreased compliance with preventive advice, and cyclic patterns of delayed care and escalating disease burden in pediatric populations worldwide [3]. Contemporary PD, therefore, emphasizes pain management as an integral part of behavior guidance whose successful management of procedural discomfort helps to improve communications and establish trust among the child, parent, and clinician and to enable the provision of comprehensive care in either private practice or community settings broadly [4]. Pharmacological strategies of local anesthesia, topical agents, analgesics, and sedation at different levels have been the mainstay of pain control in children for several decades and can be predictable in their effect if properly titrated but also elicit constant concerns regarding safety, sensitivity of technique, and acceptance in young children during procedures [5].

Parallel to pharmacological methods, nonpharmacological strategies such as tell-show-do, distraction, play therapy, parental presence, and new digital interventions have become more prominent for their ability to lessen the perception of pain and anxiety and to maintain consciousness and positive dental experiences in children across different clinical settings [6]. Recent clinical trials have tested innovative modalities, including virtual reality, audiovisual distraction, animal-assisted activities, photobiomodulation, and computer-controlled anesthetic delivery, with varying degrees of effectiveness with the modulation of pain perception, behavior, and emotional responses during common dental procedures in pediatric settings using different age groups in the world [7]. Despite this growing evidence base, heterogeneity in study designs, outcome measures, age ranges, and procedural situations means that direct comparison of interventions adds complexity to formulating consensus about optimal pain management pathways dependent on developmental stage, treatment complexity, and individual child needs based on contemporary pediatric dental practice environments that exist globally today [8].

Beyond considerations of chairside management, pain management in PD has important implications at the population level, as fear and pain that otherwise have to be managed are known barriers to service utilization with an unequal burden on children from socioeconomically disadvantaged backgrounds that contributes to persistent oral health inequalities across public health systems in many regions of the world [9]. Public health-oriented research has illustrated the importance of incorporating effective pain control mechanisms into community dental programs, school-based services, and primary care models to promote access, provide early engagement and avert the progression of dental disease among vulnerable pediatric populations in diverse socioeconomic settings [10].

Equally relevant are workforce and training considerations with the competencies required of dentists in both pharmacological and behavioral techniques, ethical decision-making, and communication skills necessary when providing safe, child-centered, culturally responsive, and adaptable to resource-constrained settings pain management within the context of modern pediatric oral health care systems [11]. Within this context, a process of systematic synthesis of contemporary evidence is essential in order to clarify relative benefits and limitations of available pain management strategies and identify best practices for routine clinical care to inform guideline development that aligns clinical effectiveness with broader public health objectives at national, regional, and global levels [12].

Existing evidence tends to be focused narrowly in terms of either modality or procedural context, resulting in gaps in integrated understanding of the interaction of different types of clinical approaches with psychosocial dimensions, access to care, and health systems functioning in determining pediatric oral health outcomes in different populations, service delivery models, and economic environments around the world [7,13]. The currently available literature demonstrates the importance of synthesizing clinical and public health evidence such that it rendezvous with the role of pain management strategies found in the pediatric dental literature, assessing the discrepancy in reported effectiveness and implications, and informing the approaches used to further the child’s oral health outcomes while addressing the lingering inequities across dental practice, policy, and dental prostheses [14].

Objectives of the review

The objectives of this systematic review are to critically evaluate clinical pain management strategies used in PD, evaluate the effectiveness of pain management strategies for pediatric dental procedures in terms of the reduction of pain and anxiety, and evaluate the implications of pain management strategies for accessing care and oral health outcomes. The purpose of this review is also to place these strategies within the context of public health approaches, which can have an important role to play in addressing equity, acceptance of treatment, and the delivery of preventive pediatric oral healthcare.

## Review

Methodology

Search Strategy

A systematic literature search was conducted in PubMed/MEDLINE, Scopus, Web of Science, and the Cochrane Library to identify studies evaluating pain management strategies in PD. The search combined controlled vocabulary (e.g., MeSH terms) and free-text keywords using Boolean operators (AND, OR). The core search string included the following: (“pediatric dentistry” OR “paediatric dentistry” OR “child dental care”) AND (“pain management” OR “pain control” OR “analgesia”) AND (“anxiety” OR “dental anxiety” OR “fear”) AND (“behavior management” OR “behavioural techniques” OR “non-pharmacological methods”) AND (“sedation” OR “conscious sedation” OR “pharmacological management”). These terms were adapted for each database.

The search was limited to studies involving human participants, published in English, between 2015 and 2025. All identified records were exported, and duplicate entries were removed before screening. Reference lists of eligible studies were manually screened to identify additional relevant articles.

Eligibility Criteria

Inclusion criteria: Studies involving pediatric patients aged 0-18 years that evaluated pain management strategies during dental procedures and reported outcomes related to pain perception, anxiety, behavioral response, or treatment acceptance were included. Randomized controlled trials and observational comparative studies were considered eligible. Both preventive and restorative dental procedures were included.

Exclusion criteria: Noncomparative studies, editorials, case reports, conference abstracts, non-English publications, animal studies, and studies lacking clearly reported outcomes related to pain or anxiety were excluded. Studies not meeting eligibility criteria after full-text assessment were excluded from the final analysis.

Study Selection

Study selection was conducted in accordance with the PRISMA guidelines. After removal of duplicates, titles and abstracts were screened for relevance, followed by full-text assessment based on predefined eligibility criteria. Reasons for exclusion at the full-text stage were documented. The study selection process is presented in the PRISMA flow diagram.

Data Extraction and Synthesis

Data were extracted using a standardized approach, including study design, sample characteristics, age range, dental procedure type, intervention and comparator, outcome measures, and key findings. Due to heterogeneity in interventions, outcome measures, and study designs, quantitative meta-analysis was not performed. Findings were synthesized narratively and grouped into pharmacological, nonpharmacological, and adjunctive/technology-assisted categories. Results were summarized in structured tables to facilitate comparison. Due to the heterogeneity in study designs, interventions, and outcome measures, quantitative synthesis and statistical pooling were not performed; therefore, statistical indicators such as p-values, CIs, and heterogeneity measures (e.g., I² statistics) were not applicable to this review.

Quality Assessment

Methodological quality was assessed based on study design, clarity of objectives, sample selection, outcome measurement reliability, and completeness of follow-up. Randomized controlled trials and observational studies were evaluated using design-specific methodological criteria. Quality assessment informed interpretation of findings; however, no studies were excluded solely based on quality.

Risk of Bias Assessment

Risk of bias was assessed at the individual study level using the Cochrane Risk of Bias (RoB) framework. Domains evaluated included selection bias, performance bias, detection bias, attrition bias, and reporting bias. For observational studies, additional consideration was given to baseline comparability and control of confounding variables. Studies were classified as low, moderate, or high risk of bias, and these assessments were incorporated into the overall interpretation of results.

Results

Study Selection

**Figure 1:**
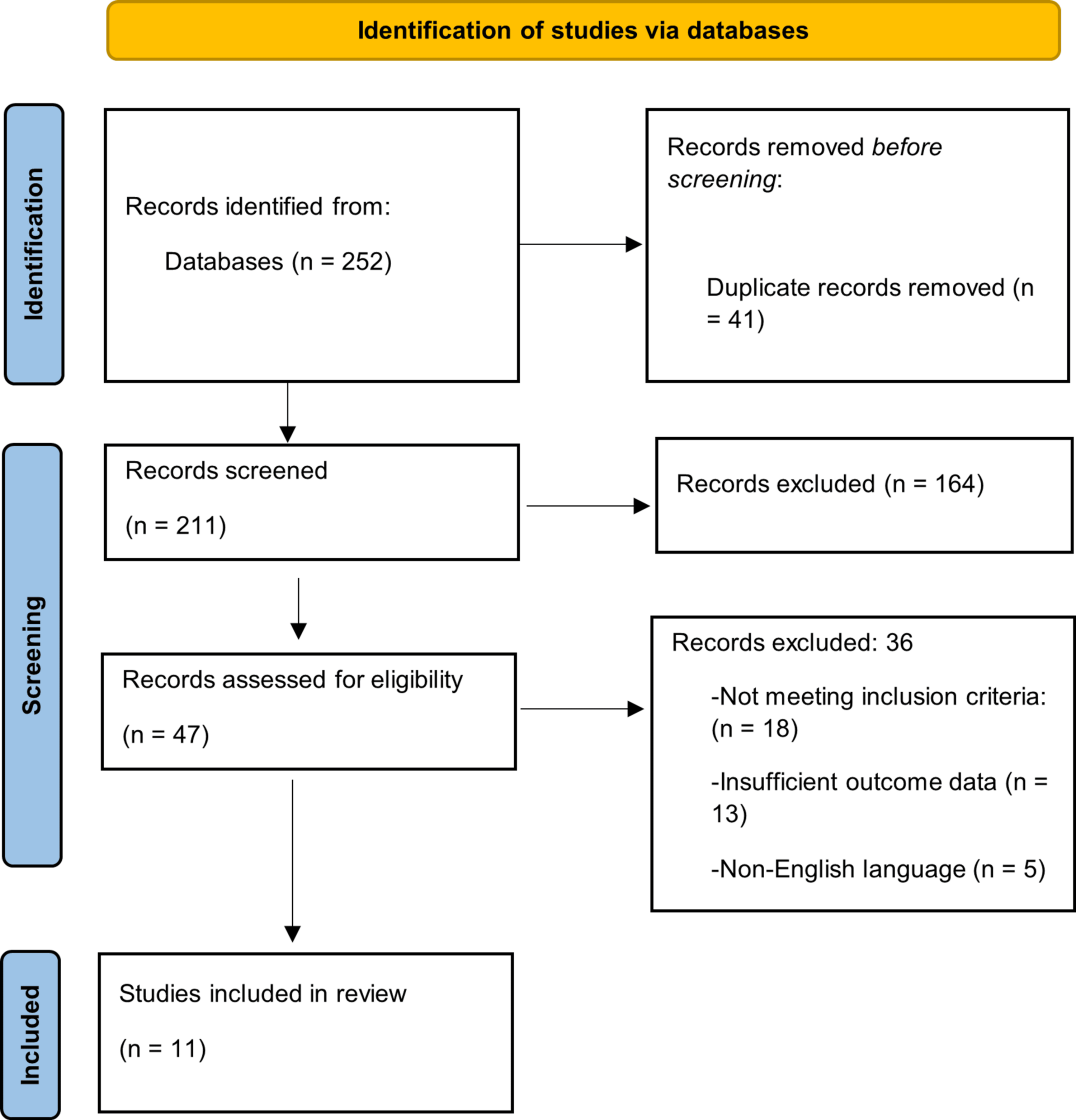
PRISMA flowchart

A total of 252 records were generated by the initial search of electronic databases. After eliminating 41 duplicate records, 211 records remained for the initial screening phase. After the screening of the titles and abstracts, 164 records were excluded as they were not relevant to the study topic. The remaining 47 full-text articles were evaluated for eligibility. During this full-text screening process, 36 records were excluded due to the following reasons: failing to meet inclusion criteria (n = 18), insufficient outcome data (n = 13), and non-English language publications (n = 5). A total of 11 studies fit all the criteria and were included in the final review. The full study selection process was carried out and reported in line with the PRISMA guidelines, and a detailed diagram of identification, screening, eligibility judgment, and inclusion is shown in Figure 1.

Study Characteristics

The included studies were randomized controlled trials or observational comparative studies that were published within the defined time period. The research was carried out in various geographic areas and clinical settings, such as private dental clinics, university hospitals, and community-based dental centers. Sample sizes were varied, and most of the participants were in the age group from preschool to early adolescence. Procedures performed on teeth that were evaluated were local anesthetic procedures, restorative procedures, extractions, pulpotomies, and preventive measures. Pain management strategies measured across studies included pharmacological, behavioral, and technological. Table 1 presents the main characteristics, clinical setting, outcome measures, and main findings of the studies included in the review.

**Table 1:** Study characteristics and key findings of pain management strategies in PD PD, pediatric dentistry

Study	Dental procedure	Study design and population	Main outcomes assessed	Key findings
Innes et al. [15]	Caries management in primary teeth	Randomized controlled trial; children in general dental practice	Pain experience, treatment acceptability, and clinical outcomes	Minimally invasive caries management approaches were associated with reduced pain and improved child acceptance compared with conventional methods.
Shetty et al. [16]	Dental treatment under local anesthesia	Randomized clinical trial; children aged 5-8 years	Pain perception, dental anxiety	Virtual reality distraction significantly reduced pain and anxiety during dental procedures.
Kolhe et al. [17]	Dental extraction	Randomized controlled study; pediatric patients	Dental anxiety levels	White, brown, and pink noise all reduced anxiety, with variations in effectiveness across noise types.
Arslan et al. [18]	Dental procedures requiring local anesthesia	Randomized clinical trial; children	Pain and dental anxiety	Lavender oil inhalation resulted in significant reductions in pain perception and anxiety compared with the control.
Shi et al. [19]	Preventive oral health education	Controlled intervention study; preschool children	Oral hygiene knowledge, oral hygiene status	Game-based interventions improved oral hygiene knowledge and indirectly supported positive dental attitudes.
Shaat et al. [20]	Sedation for dental treatment	Randomized controlled clinical trial; pediatric dental patients	Sedation efficacy, safety, and anxiety	Intranasal dexmedetomidine showed effective sedation with favorable anxiety control compared with sublingual administration.
Diab et al. [21]	Dental procedures requiring anesthesia	Clinical trial; children	Pain perception, anesthesia effectiveness	Photobiomodulation therapy demonstrated potential as an alternative to conventional local anesthetic injections.
Seraj et al. [22]	Soft tissue anesthesia reversal	Preliminary randomized clinical trial; pediatric patients	Duration of anesthesia, discomfort	Diode laser photobiomodulation accelerated the reversal of soft tissue anesthesia with reduced discomfort.
LeHew et al. [23]	Restorative dental care access	Audit study: children with Medicaid vs private insurance	Access to care, service utilization	Children with Medicaid experienced reduced access to restorative dental care, highlighting public health inequities.
Philip et al. [24]	PD training simulation	Pilot pedagogical study; dental students	Skill acquisition, perceptions	Haptic virtual reality simulation enhanced learning experiences and preparedness for pediatric dental procedures.
Elamin et al. [25]	Placement of preformed metal crowns	Randomized clinical trial; children	Pain, clinical success, and acceptability	The Hall technique was associated with lower discomfort and comparable clinical outcomes relative to conventional techniques.

Pharmacological Pain Management Approaches

Pharmacological interventions constituted a core component of pain management across the included studies and demonstrated consistent effectiveness in reducing procedural pain when appropriately administered. These approaches, particularly local anesthesia and sedation, were essential for invasive procedures and provided predictable analgesic outcomes. Variations in pain perception were associated with delivery techniques, adjunct medication use, and procedural complexity. While fewer in number compared to nonpharmacological studies, pharmacological strategies formed the foundation of pain control, with other approaches acting primarily as complementary measures. The comparative effects of various pain management strategies and their effect on pain perception and anxiety outcomes in pediatric dental care are shown in Table 2.

**Table 2:** Summary of pain and anxiety outcomes across pediatric dental pain management strategies

Pain management strategy	Type of intervention	Dental procedure context	Reported effect on pain and/or anxiety	Reference
Minimally invasive caries management	Nonpharmacological/clinical technique	Caries management in primary teeth	Reduced procedural discomfort and improved child acceptance compared with conventional approaches	[15]
Virtual reality distraction	Technology-assisted behavioral intervention	Restorative procedures, extractions	Significant reduction in pain perception and dental anxiety	[16]
Auditory distraction (noise modulation)	Behavioral distraction	Dental extraction	Decrease in anxiety levels, with variability based on auditory stimulus type	[17]
Aromatherapy (lavender inhalation)	Complementary nonpharmacological intervention	Procedures under local anesthesia	Lower pain scores and reduced anxiety compared with the control group	[18]
Sedation with dexmedetomidine	Pharmacological intervention	Dental treatment requiring sedation	Effective anxiety control with an acceptable safety profile	[20]
Photobiomodulation therapy	Adjunctive technological intervention	Dental procedures requiring anesthesia	Reduced pain perception and improved procedural comfort	[21]

Nonpharmacological and Behavioral Interventions

Nonpharmacological approaches were well represented amongst the included studies. Behavioral techniques like tell-show-do, distraction techniques, play-based intervention, and parental presence were evaluated for their effects on changes in perception of pain and also on anxiety levels. Most studies found decreased expression of observed pain behaviors and self-reported anxiety in children exposed to these techniques. Interventions involving active involvement of children, especially through the visual, auditory, or interactive modes, are more effective in enhancing cooperation during dental procedures.

Adjunctive and Technology-Assisted Strategies

A subgroup of studies assessed adjunctive and technology-based techniques such as virtual reality, audiovisual distraction techniques, laser technologies, photobiomodulation, and computer-controlled anesthetic delivery systems. These approaches showed promising results in the reduction of pain perception, as well as anxiety, especially during invasive or anxiety-provoking procedures. Technology-assisted strategies were often linked to an improvement in behavioral outcomes as well as enhanced patient acceptance; however, this was tempered by a variability in the availability of technology and protocols for implementation.

Comparative Effectiveness of Interventions

**Figure 2:**
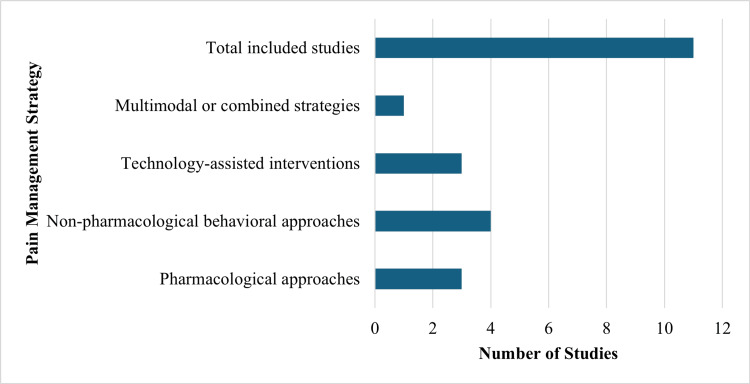
Distribution of pain management strategies among included studies

Comparative analyses indicated that pharmacological approaches remain fundamental for achieving effective pain control, particularly during invasive procedures, whereas nonpharmacological and adjunctive techniques primarily contribute to anxiety reduction and behavioral management. Although multimodal approaches demonstrated superior outcomes, these findings reflect a complementary model in which pharmacological methods serve as the primary modality, supported by behavioral and technological interventions. Figure 2 shows the distribution of pain management strategies assessed across the reviewed included studies.

Quality of Evidence and Risk of Bias Summary

Overall methodological quality varied from moderate to high for the included studies. The results of these studies showed that randomized controlled trials tended to show more rigorous reporting by way of outcomes and structured intervention protocols, as compared with the observational studies, where the control of confounding factors varied. The risk of bias assessment using RoB software revealed that most studies suffered from a low to moderate risk of bias, with common concerns surrounding blinding and outcomes assessment implemented. These quality considerations were considered in the interpretation of the findings and the strength of the evidence. The distribution of selection, performance, and detection bias among studies and the overall risk of bias assessment are presented in Table 3.

**Table 3:** Risk of bias assessment of included studies PD, pediatric dentistry

Study	Study design	Selection bias	Performance bias	Detection bias	Overall risk of bias
Innes et al. [15]	Randomized controlled trial	Low	Moderate	Low	Low
Shetty et al. [16]	Randomized clinical trial	Low	Moderate	Moderate	Moderate
Kolhe et al. [17]	Randomized controlled study	Low	Moderate	Moderate	Moderate
Arslan et al. [18]	Randomized clinical trial	Low	Moderate	Low	Moderate
Shi et al. [19]	Controlled intervention study	Moderate	Moderate	Moderate	Moderate
Shaat et al. [20]	Randomized controlled clinical trial	Low	Moderate	Low	Low
Diab et al. [21]	Clinical trial	Moderate	Moderate	Moderate	Moderate
Seraj et al. [22]	Preliminary randomized clinical trial	Moderate	Moderate	Low	Moderate
LeHew et al. [23]	Audit study	Moderate	Low	Moderate	Moderate
Philip et al. [24]	Pilot pedagogical study	Moderate	Moderate	Moderate	Moderate
Elamin et al. [25]	Randomized clinical trial	Low	Moderate	Low	Low

Discussion

Clinical Effectiveness of Pain Management Strategies

The findings indicate that pain management in PD involves pharmacological, behavioral, and technology-assisted approaches that collectively influence pain perception, anxiety reduction, and cooperation. Pharmacological methods, particularly local anesthesia and sedation, remain the primary modality for controlling procedural pain, especially during invasive procedures. Behavioral and nonpharmacological strategies act as adjuncts by addressing psychological aspects of pain, while technology-assisted interventions enhance engagement and distraction. Multimodal approaches demonstrated more favorable outcomes compared to single interventions, supporting a comprehensive and child-centered model of care.

Long-Term Behavioral and Clinical Implications

Pain management has implications beyond immediate clinical outcomes, influencing children’s long-term attitudes toward dental care [26]. Early experiences of pain and fear are associated with increased anxiety, avoidance behavior, and negative expectations in later life [27]. Effective control of pain and anxiety contributes to improved acceptance of dental care and supports consistent oral health behaviors [28].

Role of Individualized and Behavioral Approaches

No single strategy is universally optimal, highlighting the need for individualized care. Pharmacological interventions remain essential for procedures involving tissue manipulation but are more effective when combined with behavioral guidance techniques such as tell-show-do, distraction, and reassurance [4,6]. These approaches enhance understanding, predictability, and perceived control, particularly in younger or first-time dental patients [3].

Technological Interventions and Practical Considerations

Technology-assisted strategies, including virtual reality and audiovisual distraction, have demonstrated effectiveness in reducing pain perception and observable distress [2,7]. These interventions may be particularly beneficial for children with high anxiety levels or limited coping skills. However, implementation is influenced by feasibility, cost, and accessibility, which may limit their equitable adoption across clinical settings [9].

Public Health Implications

Pain and fear remain significant barriers to utilization of pediatric dental services and contribute to delayed care and progression of preventable oral diseases [10]. Integration of effective pain management strategies into community-based and primary care models may improve early engagement, treatment acceptance, and preventive care uptake, thereby reducing disease burden at the population level [1,12].

Integration of Pain and Behaviour Management

Pain and anxiety are closely interrelated, with each influencing the other [5]. Interventions addressing both dimensions simultaneously are more effective. Behavioral strategies that promote communication, trust, and positive reinforcement support a clinical environment in which pharmacological interventions can be delivered more effectively [11]. This integrated approach aligns with contemporary principles of PD that emphasize both clinical and psychological outcomes [8].

Clinical and System-Level Benefits

Effective pain management improves procedural efficiency, reduces treatment duration, and enhances patient cooperation [6]. These benefits are particularly relevant in complex clinical cases and high-demand public health settings. Reduced distress also contributes to improved caregiver satisfaction and overall quality of care [3,4].

Interpretation

Pain management in PD is a multidimensional component of high-quality care. A balanced, multimodal approach that integrates pharmacological, behavioral, and technological strategies is most effective in addressing the complex nature of pain and anxiety in children. Emphasis on individualized, developmentally appropriate, and context-sensitive care can improve clinical outcomes, patient experiences, and long-term oral health trajectories.

Limitations and Future Directions

A few limitations when interpreting the findings of this review should be noted. Considerable heterogeneity was found across included studies with respect to study design, age groups, dental procedures, and tools of pain assessment, limiting direct comparison of outcomes. Many studies were based on subjective measures of pain and anxiety, which are potentially affected by the reporting bias and developmental factors. Sample sizes were often small, and follow-up periods were often short, limiting analysis of long-term effects. Also, most of the studies were performed in a controlled clinical setting, limiting the generalizability of the findings to community or low-resource environments and routine practice settings.

Well-designed multicenter trials using standardized assessment tools to measure pain and anxiety using age-appropriate templates need to be considered in future research to enhance comparability of findings. Longitudinal studies are needed to examine the long-term consequences of early pain control from dental attendance and oral health behaviors and disease. A greater emphasis placed on community-based and public health settings would increase external validity and equity relevance. Research that will examine the cost-effectiveness, feasibility, and implementation of multimodal strategies is warranted. In addition, the perspectives of caregivers and qualitative methods may provide more information about acceptability and effectiveness in the real world across pediatric dental experiences in diverse settings, throughout the world.

## Conclusions

This systematic review draws on the current literature on pain management strategies in PD with emphasis on their clinical effectiveness and implications for wider policy on oral healthcare for children. The findings show that pain management is intrinsically multidimensional, which requires pharmacological methods, behavioral guidance, and technology-assisted interventions to be used together to consider both physiological and psychological aspects of pain. Pharmacological approaches are still integral in invasive procedures, but nonpharmacological and adjuvant strategies play an important role in minimizing anxiety, improving cooperation, and improving treatment experiences. Multimodal approaches were also consistently found to be more effective than were single techniques, emphasizing the importance of individualized and developmentally appropriate approaches to care. In addition to short-term procedural results, effective pain management is related to positive dental attitudes and better acceptance of procedures, as well as engagement with oral health services. These factors are of special relevance in pediatric populations in which early experiences influence health behaviors. The review underlines the importance of clinicians using flexible, evidence-based pain control strategies within the context of the clinical situation and the patient’s needs. The review advocates for pain management as a cornerstone of pediatric dental care to help improve outcomes, enhance experience, and contribute to oral health promotion equity.
